# Ultrasound and dynamic functional imaging in vascular cognitive impairment and Alzheimer’s disease

**DOI:** 10.1186/s12916-017-0799-3

**Published:** 2017-02-09

**Authors:** Branko Malojcic, Panteleimon Giannakopoulos, Farzaneh A. Sorond, Elsa Azevedo, Marina Diomedi, Janja Pretnar Oblak, Nicola Carraro, Marina Boban, Laszlo Olah, Stephan J. Schreiber, Aleksandra Pavlovic, Zsolt Garami, Nantan M. Bornstein, Bernhard Rosengarten

**Affiliations:** 1 Department of Neurology, University Hospital Center Zagreb, Zagreb School of Medicine, Kispaticeva 12, 10000 Zagreb, Croatia; 2 Department of Psychiatry, HUG Belle-Idée, University of Geneva School of Medicine, Geneva, Switzerland; 3 Department of Neurology, Northwestern University Feinberg School of Medicine Chicago, Chicago, IL USA; 4 Department of Neurology, São João Hospital Center and Faculty of Medicine of University of Porto, Porto, Portugal; 5 Cerebrovascular Disease Center, Stroke Unit, University of Rome Tor Vergata, Rome, Italy; 6 Department of Vascular Neurology and Intensive Therapy, University Medical Center Ljubljana, Ljubljana, Slovenia; 7 Department of Medical Sciences, Clinical Neurology-Stroke Unit, University Hospital, University of Trieste, Trieste, Italy; 8 Department of Neurology, University of Debrecen, Debrecen, Hungary; 9 Department of Neurology, Charite - Universitätsmedizin Berlin, Berlin, Germany; 10 Neurology Clinic, Clinical Center of Serbia, Faculty of Medicine, University of Belgrade, Belgrade, Serbia; 11 Methodist DeBakey Heart and Vascular Center, Houston, TX USA; 12 Neurology Department, Tel Aviv Sourasky Medical Centre, Tel Aviv, Israel; 13 Department of Neurology, Justus-Liebig-University Giessen, Giessen, Germany

**Keywords:** Vascular cognitive impairment, Vascular dementia, Alzheimer disease, Cerebral small vessel disease, Ultrasonography, Transcranial Doppler sonography, Arterial spin labelling

## Abstract

**Background:**

The vascular contributions to neurodegeneration and neuroinflammation may be assessed by magnetic resonance imaging (MRI) and ultrasonography (US). This review summarises the methodology for these widely available, safe and relatively low cost tools and analyses recent work highlighting their potential utility as biomarkers for differentiating subtypes of cognitive impairment and dementia, tracking disease progression and evaluating response to treatment in various neurocognitive disorders.

**Methods:**

At the 9th International Congress on Vascular Dementia (Ljubljana, Slovenia, October 2015) a writing group of experts was formed to review the evidence on the utility of US and arterial spin labelling (ASL) as neurophysiological markers of normal ageing, vascular cognitive impairment (VCI) and Alzheimer’s disease (AD). Original articles, systematic literature reviews, guidelines and expert opinions published until September 2016 were critically analysed to summarise existing evidence, indicate gaps in current knowledge and, when appropriate, suggest standards of use for the most widely used US and ASL applications.

**Results:**

Cerebral hypoperfusion has been linked to cognitive decline either as a risk or an aggravating factor. Hypoperfusion as a consequence of microangiopathy, macroangiopathy or cardiac dysfunction can promote or accelerate neurodegeneration, blood-brain barrier disruption and neuroinflammation. US can evaluate the cerebrovascular tree for pathological structure and functional changes contributing to cerebral hypoperfusion. Microvascular pathology and hypoperfusion at the level of capillaries and small arterioles can also be assessed by ASL, an MRI signal. Despite increasing evidence supporting the utility of these methods in detection of microvascular pathology, cerebral hypoperfusion, neurovascular unit dysfunction and, most importantly, disease progression, incomplete standardisation and missing validated cut-off values limit their use in daily routine.

**Conclusions:**

US and ASL are promising tools with excellent temporal resolution, which will have a significant impact on our understanding of the vascular contributions to VCI and AD and may also be relevant for assessing future prevention and therapeutic strategies for these conditions. Our work provides recommendations regarding the use of non-invasive imaging techniques to investigate the functional consequences of vascular burden in dementia.

## Background

With the growing burden of dementia, there is an increasing interest in identifying clinical tools that would result in early recognition of individuals at risk, differentiate between types of dementia, and eventually lead to effective prevention or treatment strategies. There is increasing evidence linking cerebral hypoperfusion and neurodegeneration, specifically in Alzheimer’s disease (AD) and vascular dementia (VaD) [1, 2]. Indeed, in the Rotterdam study [3], cerebral hypoperfusion was demonstrated to be either a risk or an aggravating factor in dementia. We also know that chronic hypoperfusion is not only an epiphenomenon of brain tissue loss but actively promotes, initiates or accelerates neurodegeneration through multiple mechanisms, including induction of oxidative stress, amyloid beta (Aβ) accumulation and aggregation, tau hyperphosphorylation, synaptic dysfunction, neuronal loss, white matter hyperintensities (WMH), and neuroinflammation [4, 5]. Vascular ageing with consequent chronic hypoperfusion may contribute to blood-brain barrier disruption which, according to emerging in vivo imaging evidence, occurs relatively early in life in selected areas, such as hippocampus, making affected individuals prone to development of memory disorders [6, 7]. A better understanding of the mechanisms underlying cerebral hypoperfusion could lead to effective therapeutic strategies targeted at treating both AD and VaD [8].

Changes in cerebral microcirculation are observed in normal ageing as well as in the broad spectrum of vascular cognitive impairment (VCI), ranging from vascular mild cognitive impairment (MCI) to VaD. Besides alterations at the level of cerebral microcirculation, which are thought to play a major role in the development of VCI, there is evidence that large vessel pathology, in particular atherosclerosis, and modifiable vascular risk factors such as hypertension, may also contribute to cognitive decline [9–11]. Ultrasonography (US) may be used to identify modifiable risk factors known to promote dementia. For instance, atherosclerotic burden, as defined by carotid ultrasound, was associated with worse cognitive performance and subsequent cognitive decline in the Tromsø and other studies [12, 13]. Cerebral microembolism is also recognised as a mechanism contributing to VCI. The negative impact of the number of microscopic emboli on cognition following surgical procedures, measured using transcranial Doppler ultrasound (TCD), has been well established in cardiac and vascular surgery procedures [14–16].

Recent studies have started to use TCD to examine the relationship between cerebral small vessel function and MRI changes attributed to small vessel disease in motor and cognitive disorders [17–22]. While TCD cannot match the spatial resolution of functional MRI or positron emission tomography (PET), it is superior to the other functional neuroimaging methods due to an excellent temporal resolution (5 ms) and it is easy to apply and robust against movement artifacts [23]. A few studies have shown a link between carotid disease and impaired cerebrovascular hemodynamics as measured by TCD [24, 25].

Arterial spin labelling (ASL), an MRI technique used to measure tissue perfusion in capillaries and small arterioles, can also be used to investigate the role of microvascular pathology and hypoperfusion in neurodegenerative disorders. ASL measures have shown hypoperfusion mainly in bilateral parietal areas and precuneus areas in MCI and mild AD patients. These same areas overlap with the patterns of hypometabolism on ^18^F-2-fluoro-2-deoxy-D-glucose (FDG)-PET in more advanced disease, hence supporting a role for perfusion monitoring with ASL as a tool to identify individuals at risk in the pre-clinical state [26, 27]. Recent work demonstrating the importance of the glymphatic system for the clearance of Aβ from the brain via the perivascular space surrounding cerebral blood vessels [28] provides additional support for altered microcirculation and functional accumulation of pathogenic macromolecules (Aβ) as an underlying mechanism contributing to neurodegeneration.

## Methods

**Fig. 1:**
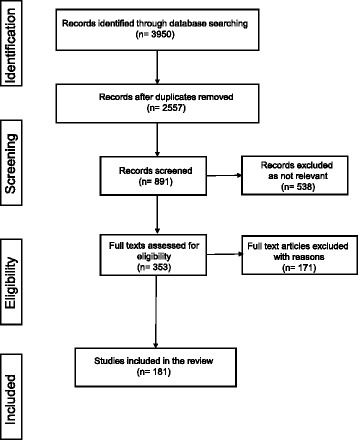
Flow diagram of the inclusion/exclusion process of the relevant literature with number of articles at each step

At the 9th International Congress on Vascular Dementia (Ljubljana, Slovenia, October 2015) a writing group of experts was formed to review the evidence on the utility of US and ASL as neurophysiological markers of normal ageing, VCI and AD. The writing group conducted a comprehensive search in Medline, PubMed and Embase databases according to the Preferred Reporting Items for Systematic Reviews and Meta-Analyses (PRISMA) guidelines 2009 [29]. The keywords vascular cognitive impairment, vascular dementia, Alzheimer disease, cognition, cerebral small vessel disease, ultrasonography, transcranial Doppler sonography, cerebral autoregulation, vasoreactivity, neurovascular coupling, microembolism, carotid ultrasound, intima media thickness, carotid artery stiffness and arterial spin labelling were used as appropriate in each database. As an example, in PubMed, the following search string was applied: ((Ultrasonography, Doppler, Transcranial[MeSH Terms] OR TCD OR Ultrasonography, Doppler, Duplex[MeSH Terms] OR “Transcranial Color-Coded Duplex Sonography” OR “Microembolic signals” OR “Arterial Spin Labelling” OR “Functional Transcranial Doppler” OR Hemodynamics[MeSH Terms] OR Carotid Intima-Media Thickness[MeSH Terms]) AND (Dementia, Vascular[MeSH Terms] OR Alzheimer Disease[MeSH Terms] OR “Vascular Cognitive Impairment” OR “Mild Cognitive Impairment” OR Cognition[MeSH Terms] OR Cerebral Small Vessel Diseases[MeSH Terms] OR Neurovascular Coupling[MeSH Terms])) AND “English”[Filter] AND “humans”[Filter]. After removing the duplicates, original articles, systematic literature reviews, guidelines and expert opinions in English published until September 2016, if considered relevant, were critically analysed to summarise existing evidence, indicate gaps in current knowledge, and, when appropriate, suggest standards of use for the most widely used US and ASL applications. A PRISMA flowchart presenting databases search results and final selection of articles is presented in Fig. 1. All members of the writing group had the opportunity to comment on the recommendations and approved the final version of this document. This review is a summary of the current state of our knowledge on this topic.

## Results

### Transcranial Doppler (TCD) and transcranial color-coded duplex sonography (TCCS)

In the 1980s, the introduction of TCD to measure cerebral blood flow velocity (BFV) [30] provided a powerful tool for non-invasive assessment of intracranial vessels. TCD, which can provide continuous beat-to-beat measurement of the BFV in the basal cerebral arteries, has become the most commonly utilised tool to study cerebrovascular haemodynamics in humans [31]. TCD can be used to non-invasively and repeatedly assess cerebral vascular responses to various physiological challenges, such as motor or cognitive activation, or changes in blood pressure and end-tidal CO_2_ regulated at the level of arterioles or resistance vessels of the brain [32–38]. Since the mechanisms underlying each of these vascular responses are likely different, they have been traditionally distinguished by different labels. Changes in BFV in response to sudden changes in blood pressure are referred to as dynamic cerebral autoregulation (CA) [32, 33], those due to changes in end-tidal CO_2_ (ETCO_2_) are referred to as cerebral vasoreactivity (VR) [34, 35], and those due to motor, visual or cognitive tasks are referred to as neurovascular coupling (NVC) or functional hyperaemia [37, 38]. Neurovascular units maintain homeostasis during periods of increased metabolic demand by coupling neural activity with cerebral blood flow (CBF). This functional hyperaemia may have slower onset or lower amplitudes if uncoupling occurs due to structural or functional damage of different elements of neurovascular units (neurotransmitters, pericytes, glia cells, vascular smooth muscle cells, endothelial cells). In addition to these three functional measures, the resting cerebral BFV waveform can also be used to calculate a pulsatility index (PI = [systolic BFV–diastolic BFV]/mean BFV), which is a measure of cerebrovascular compliance [39]. Therefore, the health of the cerebrovascular tree can easily be assessed using a neurovascular stress test battery that includes NVC, VR, and dynamic CA, as well as PI.

#### Main findings

In a recent review by Keage et al. [40], 34 articles were selected for review, of which 29 assessed TCD measures during rest, 13 during hyper/hypocapnia, and 4 employed cognitive functional TCD (fTCD) techniques (some articles presented multiple assessments). The vast majority of articles assessed differences between demented and non-demented groups rather than age-related changes (over 50 years).

A number of TCD metrics were employed to assess CBF at rest. These included mean blood flow velocity (MBFV), systolic velocity, diastolic velocity, PI and other resistance measures, and flow asymmetry index (i.e. the difference between right and left measures at rest); the majority of studies assessed the middle (MCA) or posterior cerebral arteries (PCA). Other vessels investigated included the anterior cerebral artery (ACA) and the basilar artery. Most papers assessed differences between individuals with and without dementia or dementia progression; however, some age associations in late life were reported. These studies showed that the PI increased with age [41, 42], while mean, diastolic and systolic flow velocities decreased with age in AD [43] and in the general population [41]. Most studies reported no significant differences in resting MBFV between demented and control groups [44–52], while others showed lower MBFV in demented groups, including AD [53–59], VaD or multi-infarct dementia [54, 56, 58, 60], regardless of subtype [61]. The majority of studies reported similar MBFV in AD and VaD groups [42, 48, 55, 56, 58, 60, 62], with the exception of one study, where Ries et al. [63] reported that the multi-infarct dementia group displayed lower MBFV and diastolic flow velocities compared to both control and AD groups. Caamaño et al. [58] reported that individuals with AD and multi-infarct dementia displayed reduced systolic and diastolic (as well as mean) flow velocities compared to healthy controls. The two articles which examined an MCI group assessed MBFV and reported no differences between the MCI and control groups [53, 64], unless restricting to amnestic MCI [53]. One study reported that MBFVs were more asymmetric in an AD group as compared to healthy controls [57]. In non-demented controls, lower MBFVs were associated with subsequent cognitive decline and smaller hippocampal and amygdala volumes [3]. PI was the most commonly employed measure of vessel resistance and was found to be increased in AD [47, 53–55, 60, 64] and VaD or multi-infarct dementia patients [49, 56, 60] as compared to healthy controls, although this was not the case in two papers [48, 52]. A number of reports also showed that PI was higher in individuals with VaD or multi-infarct dementia as compared to those with AD [42, 48, 52, 58]. However, in three reports [49, 56, 60], PI was not significantly different between dementia subtypes. One report comparing amnesic MCI and controls did not find a significant difference in PI [47]. Employing a different calculation of resistance (cerebrovascular resistance index = (mean blood pressure/cerebral MBFV)), van Beek et al. [44] also reported higher cerebrovascular resistance in an AD group.

#### Methodological issues

Two types of TCD equipment may be used, namely a non-duplex (non-imaging) or a duplex (imaging) device. In non-duplex devices, the arteries are identified ‘blindly’ based on the audible Doppler shift and the spectral display. Specific vessel identification is based on standard criteria, which include location of the acoustic window, orientation of the probe, depth of sample volume, direction of blood flow, and relationship with basal veins (deep middle cerebral vein for MCA and basal vein of Rosenthal for PCA), as well as on response to various manoeuvres such as the common carotid artery compression, which is not currently frequently used, and eye opening and closing [65].

TCCS imaging combines pulsed wave Doppler US with a B-mode cross-sectional view of the area of insonation, which allows identification of the arteries in relation to various anatomic landmarks. The color-coded Doppler also depicts the direction of the flow in relation to the probe while recording BFVs. In TCD, the angle of insonation is assumed to be less than 30° (as close to zero as possible) to minimise the Doppler shift measurement error. However, in TCCS, the angle of insonation can be measured and used to correct the flow velocity measurement. While TCCS significantly increases the reliability of TCD with better insonation angle correction [66], the number of studies utilising this method remains limited and most of the available data are still based on TCD.

### Functional transcranial Doppler (fTCD)

NVC (i.e. blood flow responses to neuronal activation) can be impaired as a result of neuronal or vascular pathology. fTCD is used to assess neurovascular function by measuring regional changes in cerebral BFV in response to visual, motor or cognitive challenges. fTCD is a well validated tool to assess NVC as well as hemispheric language lateralisation [67–69].

#### Main findings

A number of studies have used fTCD to show impaired neurovascular function long before the appearance of MRI surrogate measures of small vessel disease or clinical stroke symptoms in patients at a high risk of cerebrovascular injury; Fabry’s disease is one such example. In these patients, NVC in the PCA territory is impaired during a visual challenge, long before they develop clinically overt cerebrovascular disease [70]. Moreover, NVC is very sensitive to vascular risk factors and has been shown to be impaired in type 1 diabetic children [71] and in women with gestational diabetes [72]. Therefore, fTCD may be a useful pre-clinical screening tool to identify individuals at a high risk of vascular brain injury before irreversible damage occurs [73].

fTCD has also been used to assess neurovascular function in aging. Using a visual stimulation task, it has been shown that NVC in the PCA is not altered in 10- to 60-year-old healthy participants [74]. NVC in the ACA territory, however, may be affected by aging. In a study involving young and elderly subjects, NVC in the ACA territory was assessed during an executive cognitive task and compared to the PCA territory during a visual task. The results showed that NVC in the ACA territory, but not in that of PCA, was impaired in the older participants with generalisation of cerebral activity to compensate for age-related loss of region-specific function [75]. These observations suggest that NVC may be a useful biomarker of cerebrovascular aging and age-related executive dysfunction.

In addition to the potential use of NVC as a marker of vascular aging, there is promising data to suggest that NVC may also be a physiological measure of reserve or resilience. In a cohort of older community dwelling participants, intact NVC was associated with preserved mobility despite a high burden of cerebral microvascular disease by MRI [21]. Further, NVC has also been linked to cognitive function and white matter structural integrity [76]. Therefore, NVC may be a promising therapeutic target to enhance network connectivity, mobility and cognitive function. In fact, there is already promising data to show that NVC is sensitive to acetylcholinesterase inhibitors [77] and flavanols [76].

#### Methodological issues

The cerebrovascular tree should be fully assessed using cervical and TCD ultrasound to exclude hemodynamically significant stenosis in the target territory by fixing monitoring probes in an appropriate headframe. Common standard procedures include simultaneous bilateral PCA or left PCA plus control right MCA insonation settings for visual stimuli [70, 78–80] and bilateral MCA for motor, sensory, cognitive and language tasks [81, 82]. Some studies have also monitored ACA during cognitive tasks [83]. Proper headframe and stimulus signals recorded synchronously with BFV are necessary for accurate measures. Appropriate duration of resting and stimulation phases would depend on the chosen stimulus paradigm. In general, several cycles of rest/activation are averaged to increase the accuracy of the test.

Different stimulus paradigms have been used depending on the aim of the study. MCAs have been monitored during motor, sensory and cognitive tasks [81, 84–87], ACAs have mainly been recorded during cognitive tasks [82], while PCAs have been monitored during reading, flickering light, black/white pattern and checkerboard stimuli [88].

Collected data may be analysed using a number of different approaches. Some Doppler systems contain a software package for this purpose. Alternatively, there are a number of free or commercial software packages designed to simplify data analysis (dopOSCCI, AVERAGE) [84, 88]. The most commonly analysed parameter is the (relative) evoked BFV response (EFR), which is calculated as EFR = [(maximum BFV – resting pre-test BFV)/resting pre-test BFV] × 100%; it is also commonly referred to as the overshoot parameter. A more complex analysis of the dynamic aspects of the visual evoked BFV response has been developed, described by a control system analysis (parameters of time delay, rate time, natural frequency, attenuation and gain) [78–80, 88].

#### Limitations

General limitations of all TCD methods are the absence of an acoustic window, which limits the use in a small percentage of patients and provides low spatial resolution, i.e. examination is limited to vascular territories of the circle of Willis. In patients with significant oscillations in the cardiac cycle (such as atrial fibrillation) due to oscillations in BFV, the measured values should be corrected by averaging the activity within a single heart cycle.

#### Potential applications

fTCD can be used as a pre-clinical screening tool to identify individuals at a high risk of vascular brain injury or to detect early neurovascular dysfunction with ageing or in high risk patients. Another interesting application may be to monitor for the therapeutic effects of different drugs on NVC.

### Cerebral vasoreactivity (VR) tests

The response to vasodilatory stimuli, such as CO_2_ or acetazolamide [89–92], has been traditionally used to evaluate and quantify cerebral VR, also referred to as cerebrovascular or vasomotor reactivity. While the exact mechanisms are unknown, these agents dilate the cerebral arterioles and therefore increase CBF [93–95]. Some have argued that this vasodilatory response may be a measure of endothelial function in the cerebral vessels [96]. Cerebral VR is assessed from BFV responses to rapid changes in ETCO_2_. The increase in partial pressure of CO_2_ induces dilation of cerebral arteries and arterioles, resulting in a cerebral BFV increase, while a reduction in partial pressure of CO_2_ results in vasoconstriction and BFV decrease. CO_2_-induced cerebral VR can be measured either by direct administration of CO_2_ or with the breath holding index (BHI); there is extensive literature reporting both measures in studies of cognition. On the other hand, only two studies reported using acetazolamide and TCD for VR testing in patients with different types of dementia [45, 62].

#### Main findings

One of the first studies to pave the way in testing the link between VR and cognition was based on the comparison of VR across controls and patients with dementia subtypes, and found a marked reduction of vasodilatory reserve in VaD patients compared to AD patients and controls [97]. Nevertheless, a more recent study has not been able to show that VR can differentiate between dementia subtypes [56].

Further studies in fact showed that cognitive function is well correlated with BHI in AD patients [98], independently of the presence of microvascular ischemic MRI lesions [55] and with a cut-off value of BHI < 0.69 as a possible predictive marker of MCI conversion to AD [99]. Other studies also showed that VR assessed by BHI is significantly reduced in the AD compared to MCI and healthy controls [100, 101]. These findings are further supported by a recently reported association between reduced VR and the APOE ε4+ genotype in MCI patients with increased susceptibility to developing dementia [102]. Therefore, BHI may be a useful biomarker to identify MCI subjects at high risk of AD conversion [101]. Moreover, impaired VR is also associated with an unfavourable evolution of cognitive function in AD patients, suggesting that BHI could also be a valuable marker for disease progression [98, 103, 104].

Finally, in a cohort of subjects with asymptomatic carotid stenosis, cerebral VR assessed by BHI has also been shown to be a strong predictor of cognitive decline [105]. VR assessment using BHI may be a useful biomarker to identify patients with asymptomatic carotid stenosis at risk for cognitive decline who may benefit from early surgical revascularisation to prevent further cognitive decline [24].

#### Breath holding index (BHI)

Voluntary breath holding leading to hypercapnia can be used to evaluate vascular reactivity [106]; the procedure requires a collaborative participant. The BHI has been validated in patients with internal carotid artery stenosis or occlusion, compared to other neuroimaging techniques, as a marker of hemodynamic insufficiency and increased stroke risk [107].

Participants should first be fully trained to perform the procedure correctly by holding their breath for 30 s (24 s minimum) after a normal inspiration. They are connected to a respiratory activity monitor to measure the exact duration of apnea. Once they are trained, MBFV at rest is obtained by averaging flow velocities over a 1-min period of continuous recording at room air. Participants are instructed to hold their breath. After breath holding is initiated, MBFV is recorded over a 4-s interval. ETCO_2_ at rest and during the first exhalation after apnea is then evaluated by a capnometer. Outside a controlled setting, BHI can still be performed as a screening test without a respiratory activity monitor and capnometer, but the results are less reliable.

BHI is calculated by dividing the percent of increase in MBFV occurring during breath holding by the length of time (in seconds) during which the participant held their breath after a normal inspiration: BHI = [({MBFV at the end of breath holding − MBFV at rest}/MBFV at rest) × (100/time of breath holding)]. The procedure should be repeated after a resting period of normal breathing for 3 minutes, and the mean of the two results is considered for further analysis.

#### CO_2_ reactivity test

For this procedure, bilateral MCA BFVs are recorded while ETCO_2_ is changed. ETCO_2_ can be changed by asking subjects to breathe through a standard non-rebreathing mask with two one-way valves and with an incorporated 2-L reservoir bag; the circuit is connected to a capnometer that continuously measures ETCO_2_. Alternatively, the percent change in MBFV that occurs during breathing an air-CO_2_ mixture containing 5–8% CO_2_ is measured through a non-return valve connected to a mask [108]. Another variation of this approach is to follow this step with gentle hyperventilation to reduce ETCO_2_. To calculate VR, MBFV or percent change in MBFV is plotted (y-axis) against ETCO_2_ (x-axis); the slope of the linear relationship between MBFV and ETCO_2_ is VR [109]. One can also calculate a vasodilatory range by measuring the difference between maximum percent change in MBFV during CO_2_ inhalation and hyperventilation. A mean vasodilatory range of 88 ± 16% has been reported in normal individuals [107].

#### Methodological issues

VR assessment is feasible in most patient populations and clinical settings. Before proceeding with the VR tests, a complete extracranial and intracranial examination should be performed to exclude the presence of a hemodynamic stenosis that may influence the test results. Since the baseline values may also affect the vasodilatative or vasoconstrictive responses, only normocapnic participants should be included. All participants should abstain from alcohol and caffeine-containing beverages and smoking for at least 24 h prior to the study due to the possible influence on vascular reactivity. The examination should be carried out in a quiet room.

Bilateral simultaneous BFV recordings of MCAs are obtained using a TCD system with two 2-MHz transducers fitted on a headband and placed over the temporal bone window. While the MCAs have been traditionally assessed using this approach, ACA or PCAs can also be tested, if needed.

#### Limitations

The limitations associated with VR measurements are the same as the general limitations of all TCD methods (See fTCD section). In addition, the BHI technique is highly dependent on operator expertise and participant collaboration. To minimise variability, participants should be trained to perform the procedure correctly, a capnometer should be used to check the proper execution of the apnea/breath holding, and the mean value of at least two tests should be used. There is a false notion that breathing the air mixture with CO_2_ could be uncomfortable for patients. However, since the O_2_ content of the gas mixture is the same as room air, with CO_2_ simply replacing N_2_, there is no discomfort to the participant.

#### Potential applications

While at the present time, BHI is not able to discriminate among different types of dementia, it may be a useful biomarker for identifying individuals at risk of cognitive decline. Given the link between impaired VR and cognitive impairment or disease progression, VR may be a promising therapeutic target for the prevention and treatment of cognitive disorders.

### Cerebral autoregulation

A variety of measures have been used to assess the integrity of CA, but the advantages of TCD have made it the most commonly utilised tool to study CBF regulation in humans. Assessment of dynamic CA is based on transient changes in CBF in response to sudden and rapid changes in mean arterial pressure (MAP). Sudden changes in MAP can be induced using a variety of techniques such as the rapid deflation of bilateral thigh-cuffs [110], hand grip [111], Valsalva manoeuvre [33, 112, 113], transient hyperaemic response test [114, 115], deep breathing, and the sit-to-stand method [116]. The blood flow response is then characterised and quantified by a number of derived variables and reported as specific measures of autoregulation [117]. Alternatively, spontaneous oscillations in arterial blood pressure can be used to calculate the auto- and cross-spectra of and between arterial blood pressure and CBF velocity [118–122].

#### Main findings

CA differs from the already described traditional measures of VR, namely cerebral VR or vasomotor reactivity. It is important to remember that, although an intact endothelium may be necessary for adequate pressure regulation, traditional VR tests do not detect changes in cerebrovascular resistance in response to perfusion changes. Therefore, cerebral VR and CA test different properties of the cerebrovascular system and should not be used interchangeably [89, 123, 124].

Impairments in CA occur in a number of conditions such as stroke, subarachnoid haemorrhage, postpartum angiopathy, eclampsia, syncope, and traumatic brain injury. Early evidence for impairment of CA in AD came from animal studies [125]. More recently, data to link measures of CA to radiographic markers of cerebral hypoperfusion and ischemic injury as well as amyloid burden in humans has become available [126–128]. A study of 48 older individuals showed a strong relationship between impaired CA and loss of cerebral white matter structural integrity (WMH and diffusion tensor imaging metrics) [126]. Similarly, in 14 non-demented elderly individuals, reduced CA was associated with both increased amyloid deposition and increased WMH volume [127]. Few studies have examined CA in patients with AD, but the results remain inconclusive and studies were limited by their small sample size [128]. Therefore, now that the link between CA, cerebral hypoperfusion and amyloid deposition has been established, it is time to initiate more robust studies of CA in cognitive impairment and explore the role of CA in dementia subtypes.

#### Methodological issues

Similar to the VR and NVC test, a complete extracranial and intracranial examination of the cerebrovascular system should be performed to exclude any hemodynamically significant steno-occlusive disease that may influence the results. Only normocapnic subjects should be considered and ETCO_2_ should be monitored during the study to exclude any significant changes that may influence the results.

Participants are instrumented with three-lead ECG for continuous heart rate recording. Beat-to-beat arterial blood pressure recordings are obtained using a finger photoplethysmographic cuff. The Finometer transducer has to be calibrated to height. A 2-MHz TCD transducer is placed on each temporal acoustic window to measure the right and left beat-to-beat MCA BFV. The MCAs are insonated and recorded at a depth of 50–60 mm using a probe fixation device. All measurements are obtained continuously for 5 minutes while subjects are seated upright in a chair. This same approach can be applied to the ACA, PCA or combination of vessels, and is not limited to the MCAs.

Frequency-domain transfer function analysis is a valid method for examining dynamic CA (for a detailed description please see Meel-van den Abeelen [129] and Claassen [130]). Using this approach, the relationship between the spontaneous beat-to-beat fluctuations in MAP and MBFV is quantified by gain, phase and coherence of the two oscillating waveforms. The phase shift reflects the temporal difference between transmissions of oscillation from MAP to MBFV. If the oscillations of MBFV and MAP are almost synchronous, as in impaired autoregulation, the phase shift would approach zero, while higher phase shift represents better autoregulation. Gain, on the other hand, is the magnitude of transmission of MAP oscillations to MBFV. Effective autoregulation dampens the transmission of low frequency MAP oscillations onto MBFV. Therefore, a lower gain in the low frequency is interpreted as effective autoregulation. The coherence varies between 0 and 1, and is similar to a correlation coefficient; it expresses the fraction of MBFV signal that is linearly associated with MAP in the frequency domain.

#### Limitations

The limitations associated with CA measurement are the same as described for VR and fTCD.

#### Potential applications

Dynamic CA may be used to monitor responses to drugs directed at brain amyloid clearance as well as assessing and monitoring vascular contributions to neurodegeneration.

## Extracranial ultrasound

### Carotid diameter, blood flow velocity, flow and resistance

#### Main findings

Mean common carotid artery (CCA) diameter and increased PI are the only two ultrasound parameters that have been independently associated with WMH score after adjusting for demographic characteristics and vascular risk factors [18]. Studies have shown that the pulsatility of the internal carotid artery (ICA) is higher in patients with WMH than those without [131–134].

An increased or normal systolic velocity and decreased ICA diastolic velocity has also been commonly described in association with WMH [18, 133, 134]. A recent study showed decreased BFV and blood flow at all stages of the hemodynamic cycle in patients with WMH compared to the risk factor-matched control group [135]. Aribisala et al. [134] found a correlation between lower ICA diastolic BFV as well as diastolic blood pressure and WMH. These findings suggest diastolic hypoperfusion as a possible underlying mechanism of WMH. Furthermore, Schreiber et al. [136] described reduced global CBF and prolonged cerebral circulation time in patients with VaD as well as in AD compared to controls. Authors suggested that multimodal ultrasound evaluation could help assess the extent of changes in global cerebral hemodynamics in patients with dementia but does not allow for a differentiation between VaD and AD.

#### Methodological issues

The patient is usually examined in a supine position with the neck extended and the head inclined by 30^o^. A high frequency linear probe (7 MHz or higher) is used for the standard colour-coded duplex sonography (B mode, colour and pulse Doppler method). Bilateral CCA, carotid bulb, ICA and vertebral arteries should be examined in both transverse and longitudinal planes.

Carotid artery diameter measurements are performed in the longitudinal plane, strictly perpendicular to the ultrasound beam, with both proximal and distal walls clearly visualised. The diameter is usually defined as the distance between the proximal and distal intimal layer. Peak-systolic and end-diastolic BFV in all arteries are measured.

PI can be calculated from the same formula used for TCD (see TCD section) and the resistivity index can be calculated using the following formula: RI = peak systolic velocity – end diastolic velocity/peak systolic velocity). Cerebral blood flow can be calculated using the MBFV and diameters of both ICAs and vertebral arteries (CBF = Σ(MBFV × Π × [diameter/2]^2^)).

#### Limitations

Analysis may be difficult to perform if a patient has a short contour of the neck. In cases where CCA bifurcation is positioned high in the neck it may be difficult to visualise the ICA.

### Carotid plaque evaluation

#### Main findings

The relationship between WMH and unstable plaques suggest that, in addition to atherosclerosis in the cerebrovascular tree and global hypoperfusion, cerebral embolism may also contribute to cerebral vascular injury, white matter damage and neuronal loss. Instability of plaques as determined by ultrasound strain imaging also correlates with WMH [137]. Carotid intima media thickness (IMT) and plaque burden are also positively correlated with increased WMH lesion burden [138–140]. Therefore, it is possible that changes in microvasculature similar to carotid pathology and unstable plaques also contribute to development of WMH.

#### Methodological issues

Carotid IMT is defined as the maximum distance between the characteristic echoes from the lumen–intima and media–adventitia interfaces on the far wall of the common carotid artery (Mannheim criteria [141]). If present, carotid plaques (focal structure that encroaches into the arterial lumen of at least 0.5 mm or 50% of the surrounding IMT value or demonstrates a thickness > 1.5 mm) should be evaluated in the axial and longitudinal planes.

#### Limitations

Calcium deposits in the wall of the carotid artery may make it difficult to evaluate the vessel. A small amount of soft plaque producing low-level echoes may also go undetected.

### Arterial stiffness evaluation

#### Main findings

Several studies have shown that aortic stiffness determined by carotid–femoral pulse wave velocity correlates with the development, progression and degree of WMH [131, 142, 143]. According to one theory, increased large artery stiffness causes small vessel disease by impaired damping of the arterial waveform and reduced wave reflection at the interface of large and small arteries, resulting in small vessel wall damage due to increased flow pulsations [144, 145]. A recent study on 549 stroke and dementia-free middle-aged individuals has shown that pulse pressure is associated with brain atrophy and cognitive decline [146]. The study indicates that arterial stiffening, indexed by simple pulse pressure, may play a role in early cognitive decline and brain atrophy in mid-to-late life, particularly among APOE-ε4 carriers [146].

Although carotid–femoral pulse wave velocity is still considered the ‘gold standard’ for the evaluation of arterial stiffness, local carotid stiffness parameters have also been increasingly used [147]. In addition to being easily applicable and widely available, local carotid stiffness parameters show some specific characteristics [148]. For example, carotid stiffness parameters were found to be increased in patients with WMH compared to matched controls [149, 150]. The most powerful carotid stiffness parameters were pressure strain elasticity modulus and local pulse wave velocity index beta [149].

#### Methodological issues

Carotid arterial stiffness measurements are performed in the same way as IMT; the carotid artery is visualised in the longitudinal plane, strictly perpendicular to the ultrasound beam, with both walls clearly visualised. Arterial stiffness is usually measured automatically from the changes in the carotid arterial diameter between the systolic and diastolic phases on CCA segments. Carotid diameter waveforms are converted to carotid pressure waveforms using an empirically derived exponential relationship between pressure and arterial cross-section. The derived carotid pressure waveform is calibrated to brachial end diastolic and mean arterial pressure by iteratively changing the wall rigidity coefficient [151]. This allows the calculation of the arterial stiffness indices of beta stiffness, pressure-strain elasticity modulus [147, 152], arterial compliance [152], pulse wave velocity [153], and augmentation index [152].

#### Limitations

Carotid stiffness evaluation may be difficult to perform if a patient has a short neck or in cases where the CCA lies deep within the tissues and cannot be clearly visualised. A patient’s inability to lie still also makes the evaluation difficult.

#### Potential applications

Potential applications of different extracranial ultrasound methods and parameters are presented in Table 1.

**Table 1:** Potential applications of extracranial ultrasound in vascular dementia (VaD) and Alzheimer’s disease (AD)

Carotid ultrasound method	Finding	References
Aortic stiffness (carotid–femoral pulse wave velocity)	Increased aortic stiffness in VaD	Poels et al., 2012 [142] Mitchell et al., 2011 [131] Webb et al., 2012 [132]
Carotid stiffness index	Greater CCA stiffness indexes in VaD	Morovic et al., 2009 [180] Jurasic et al., 2009 [181] Turk et al., 2015 [149]
Carotid pulsatility and resistance index	Higher ICA pulsatility in patients with WMH	Mitchell et al., 2011 [131] Webb et al., 2012 [132] Tanaka et al., 2009 [133] Aribisala et al., 2014 [134]
Carotid diameter	CCA diameter increase in VaD	Morovic et al., 2009 [180] Heliopoulos et al., 2012 [18]
Carotid BFV	Higher ICA pulsatility and lower BFV in VaD patients	Tanaka et al., 2009 [133] Aribisala et al., 2014 [134] Heliopoulos et al., 2012 [18]
Cerebral blood flow	Lower CBF in VaD and AD patients compared to controls Longer CCT in VaD and AD patients compared to controls	Schreiber et al., 2005 [136]
	Similar CBF in VaD and AD patients compared to controls	Turk et al., 2015 [149]
Plaque instability: Ultrasound strain imaging	Increased strain in plaque correlated with WMH	Berman et al., 2015 [137]
Intima media thickness and plaque burden	Positive correlation between intima media thickness and plaque burden with WMH lesion burden	Casado-Naranjo et al., 2015 [138] Heliopoulos et al., 2012 [18] Kearney-Schwartz et al., 2009 [182] Shrestha et al., 2009 [140]

*BFV* blood flow velocity, *CBF* cerebral blood flow, *CCA* common carotid artery, *ICA* internal carotid artery, *WMH* white matter hyperintensities, *CCT* cerebral circulation time

### Detection of microembolic signals (MES)

Patients with silent brain infarcts have increased risk of dementia and a steeper decline in cognitive function than those without such lesions [154]. One possible mechanism leading to silent infarcts may be cerebral microembolisation. The ability of TCD to detect solid and gaseous asymptomatic microemboli can be used to identify sources of such emboli in patients at risk (right-to-left shunts, high risk atherosclerotic plaques, vascular or cardiac surgeries). Early interventions to prevent silent microembolisation may be useful in preventing silent infracts and subsequent cognitive decline.

#### Main findings

Purandare et al. [155] assessed the relationship between spontaneous resting MES and dementia and showed that the presence of MES was significantly associated with VaD. In the same large cohort, the group went on to show a higher incidence of emboli in both VaD (37% of cases) and AD (40% of cases), as compared to controls (12% of cases) [156]. Moreover, they showed that the presence of MES was not only associated with higher incidence of depression symptoms in patients with dementia, but that MES were associated with greater cognitive decline among controls (i.e. non-demented cases at baseline) [157]. More recently, the group also showed MES to be associated with faster cognitive and functional decline in AD and VaD, as well as an increased number of psychiatric symptoms over a 24-month period [158].

#### Methodological issues

The influence of US system settings on the detectability of MES is extremely important and the setup should be standardised in order to make studies relevant [159]. The methodology for detecting MES was established and defined by the International Consensus Group on Microembolus Detection in 1997 [160], which suggested that all future studies should report microemboli detection protocols uniformly. Automated detection protocols can be time saving, but their sensitivity and specificity still fail to meet requirements for routine clinical use.

#### Limitations

The time commitments required to perform the examination and analyse the results can limit the wider application of methods in daily practice. Appropriate MES recognition requires significant expertise to minimise operator variability and to exclude artifacts which are not always clearly distinguishable.

#### Potential applications

Detection and quantification of microemboli in patients at high risk of developing cognitive decline and dementia. Improvement of potentially harmful interventional techniques by reduction of microembolic signals [15, 161].

### Arterial spin labelling (ASL)

ASL is an MRI technique that allows brain perfusion to be measured non-invasively at the tissue level. Although the principle of ASL was introduced in the early 1990s, it has since significantly benefited from the improved signal-to-noise ratio of modern high-field MRI systems. The increasing availability of 3T scanners as well as the development of improved pulse sequences and multi-channel receiver array coils has led to a rapidly growing interest in ASL within the past few years, mainly in neurological and psychiatric disorders. As a complement to classic ASL, a dynamic ASL method was also proposed to assess intracranial vascular compliance in brain aging and dementia [162].

#### Main findings

Since the widespread use of whole-brain, high signal-to-noise ratio ASL sequences, several studies have shown changes in ASL parameters, primarily mean transit time and time-to-peak of the residue function, in cerebrovascular diseases [163, 164]. Vessel-selective ASL is sensitive to perfusion changes induced by collateral flow in stroke patients. It has also been applied to cases where treatment was initiated on the basis of the presence of collateral flow [165]. A widespread decrease in CBF has been found in VaD cases, especially in bilateral frontal and parietal areas but also in individuals with confluent incidental white matter ischemic changes [166] and post-stroke patients [167].

In clinically overt AD, ASL studies have consistently shown a reduction in CBF in a posterior parietal distribution, including the precuneus, posterior cingulate, angular gyrus, and superior parietal gyrus [168, 169]; for a recent review see Montagne et al. [170]. The ASL pattern is similar to that of FDG-PET hypometabolism and both modalities have a similar diagnostic performance [171, 172]. Moreover, ASL patterns seem to follow the progression of cognitive decline, suggesting that this imaging modality may also be a promising biomarker of disease progression. However, young at-risk cases may display transient hyperperfusion due to an uncoupling between CBF and local metabolic needs during different phases of the disease. Recently, the capillary heterogeneity transition time was proposed as a reliable marker of the biphasic nature of CBF responses in preclinical AD [173].

Most importantly, ASL imaging, performed at baseline, demonstrated decreased perfusion in the posterior cingulate cortex in elderly individuals who later developed subtle cognitive decline. Interestingly, the ASL pattern was similar between MCI cases and ‘normal’ controls who subsequently developed cognitive decline. These findings suggest that ASL may be a valuable predictive tool for identifying individuals in the preclinical state who have reduced CBF and are at a high risk for disease progression and cognitive decline despite an ‘intact’ cognitive status [174].

#### Methodological issues

ASL is based on magnetically labelling inflowing arterial blood protons prior to their entry into the tissue of interest. As such, it can be viewed as a tracer technique with water acting as the natural endogenous tracer to estimate tissue perfusion. The label is created by applying radio-frequency pulses to invert the bulk magnetisation of water protons in blood. Images are acquired after the labelling and inflow period using rapid acquisition techniques such as echo-planar imaging, gradient and spin echo imaging, or three-dimensional fast spin-echo imaging using a stack-of-spirals approach [175]. A pair of images is always acquired, namely a labelled image, in which the blood water magnetisation is inverted, and a control image, in which the blood water magnetisation is not inverted. The signal difference between labelled and non-labelled images is proportional to the amount of magnetisation inverted and delivered to the tissue. If all the labelled blood has arrived at the imaging voxel at the time of image acquisition, the signal difference will be proportional to CBF. The current main implementations of ASL are pulsed and pseudo-continuous labelling (which guarantees higher signal-to-noise ratio). The use of background suppression and segmented three-dimensional readout without vascular crushing gradients is also recommended mainly in cases with cognitive deficits in order to avoid significant artifacts [176]. While some of these artifacts may decrease the diagnostic value of ASL by degrading the images or mimicking pathology, others are of clinical utility because they increase the probability of AD pathology [177].

#### Limitations

Due to the low perfusion levels and long arterial arrival times in white matter, the signal-to-noise ratio of the ASL signal is typically very low. Thus, it is not possible to obtain reliable estimates of CBF in the deep white matter unless the acquisition parameters are optimised [178]. Therefore, ASL imaging is not as sensitive to perfusion changes in the deep white matter as in the cortex.

Several studies have shown fair to good concordance of ASL with measures of perfusion deficit, primarily time-based parameters such as the mean transit time and time-to-peak of the residue function [163]. Vessel-selective ASL is more sensitive in the identification of perfusion changes induced by collateral flow in stroke patients. ASL may also be useful in situations where treatment is initiated on the basis of the presence of collateral flow patterns [165].

#### Potential applications

Since MRI is part of routine workup of cognitive decline in many clinical and research settings, ASL is a cost-efficient and operator-independent tool for the assessment of early cognitive decline, which simply prolongs an already existing assessment for a few minutes. ASL could be useful as a predictive preclinical AD marker allowing for the identification of healthy controls at risk for AD dementia. Due to the known coupling between brain perfusion and metabolism, ASL may be used as a surrogate marker for FDG-PET. In the future, ASL could be combined with amyloid and tau PET imaging in the identification of healthy individuals with an already active AD process.

## Conclusions

Diagnostic tools that can provide real-time functional assessment of the cerebrovascular tree will have a significant impact on our understanding of the vascular contribution to neurodegeneration at different stages of cognitive decline. A large body of evidence is now emerging to show the utility of TCD and carotid ultrasound in providing rapid assessment of cerebrovascular haemodynamics. The biggest advantage of these US techniques is their excellent time resolution, wide availability and relatively low cost.

Emerging studies highlight the potential contribution of VR to early detection of high-risk MCI patients who will develop VaD or AD and of patients with carotid stenosis at risk of cognitive decline [99–102]. Similarly, a growing number of publications have started to examine NVC and fTCD in vascular ageing and as a measure of physiological reserve [73–75]. Other emerging studies are beginning to link impaired CA, increased CCA diameter and PI to MRI measures of cerebral small vessel disease such as WMH and white matter microstructural integrity using diffusion tensor imaging [126, 133–135, 137]. In fact, a recent study linking impaired CA to amyloid deposition provides the most promising mechanistic link between vascular disease and neurodegeneration [127].

However, we have only begun to scratch the very tip of this iceberg and TCD and US technology as tools to study vascular contributions to dementia are at their infancy. The technological aspect of US devices has not changed in recent years, but there is a constant development of new analytical tools enabling a better understanding of microvascular effects on blood flow in standardly insonated large arteries [179]. There is a significant need for a greater application of these tools across the spectrum of neurodegenerative disorders. We need to test these tools in large longitudinal epidemiological studies, smaller mechanistic studies and therapeutic clinical trials testing various interventions.

Similar studies are also emerging to show the application of ASL in assessing vascular function at the microvascular and capillary level, while providing detailed spatial resolution in patients with dementia [168–170]. Analogous to US studies, ASL studies suffer from small numbers and inadequate sample sizes. The immediate clinical utility of ASL and US also remains quite limited because most measures lack validated cut-off points to distinguish between normal and pathological cases.

This review of evidence and suggested standards for the performance of examinations aims to raise awareness of the promise that these tools hold as preclinical markers of vascular pathology in neurodegeneration and their utility in assessing disease progression and/or response to therapy. Another aim is to encourage more investigators to apply these tools and measures in their studies of neurodegeneration and to thus increase the pool of such publications. Therefore, we strongly encourage the application of US and ASL to evaluate patients with vascular risk factors who are at risk of cognitive decline and/or conversion to dementia.

## References

[1] Luckhaus C, Flüb MO, Wittsack HJ, Grass-Kapanke B, Jänner M, Khalili-Amiri R, Friedrich W, Supprian T, Gaebel W, Mödder U, Cohnen M (2008). Detection of changed regional cerebral blood flow in mild cognitive impairment and early Alzheimer’s dementia by perfusion-weighted magnetic resonance imaging. Neuroimage.

[2] Fleisher AS, Podraza KM, Bangen KJ, Taylor C, Sherzai A, Sidhar K, Liu TT, Dale AM, Buxton RB (2009). Cerebral perfusion and oxygenation differences in Alzheimer’s disease risk. Neurobiol Aging.

[3] Ruitenberg A, den Heijer T, Bakker SL, van Swieten JC, Koudstaal PJ, Hofman A, Breteler MM (2005). Cerebral hypoperfusion and clinical onset of dementia: the Rotterdam Study. Ann Neurol.

[4] Zlokovic BV (2011). Neurovascular pathways to neurodegeneration in Alzheimer’s disease and other disorders. Nat Rev Neurosci.

[5] Iadecola C (2013). The pathobiology of vascular dementia. Neuron.

[6] Montagne A, Barnes SR, Sweeney MD, Halliday MR, Sagare AP, Zhao Z, Toga AW, Jacobs RE, Liu CY, Amezcua L, Harrington MG, Chui HC, Law M, Zlokovic BV (2015). Blood-brain barrier breakdown in the aging human hippocampus. Neuron.

[7] Yang T, Sun Y, Lu Z, Leak RK, Zhang F. The impact of cerebrovascular aging on vascular cognitive impairment and dementia. Ageing Res Rev. 2016. Ahead of print. 10.1016/j.arr.2016.09.007 PMC5250548 27693240

[8] Zhao Y, Gong CX (2015). From chronic cerebral hypoperfusion to Alzheimer-like brain pathology and neurodegeneration. Cell Mol Neurobiol.

[9] van Oijen M, de Jong FJ, Witteman JC, Hofman A, Koudstaal PJ, Breteler MM (2007). Atherosclerosis and risk for dementia. Ann Neurol.

[10] Bakker FC, Klijn CJ, Jennekens-Schinkel A, Kappelle LJ (2000). Cognitive disorders in patients with occlusive disease of the carotid artery: a systematic review of the literature. J Neurol.

[11] Iadecola C (2014). Hypertension and dementia. Hypertension.

[12] Arntzen KA, Schirmer H, Johnsen SH, Wilsgaard T, Mathiesen EB (2012). Carotid artery plaque progression and cognitive decline: the Tromsø study 1994-2008. Eur J Neurol.

[13] Zeki Al Hazzouri A, Vittinghoff E, Sidney S, Reis JP, Jacobs DR, Yaffe K (2015). Intima-media thickness and cognitive function in stroke-free middle-aged adults: findings from the Coronary Artery Risk Development in Young Adults Study. Stroke.

[14] Russell D, Bornstein N (2005). Methods of detecting potential causes of vascular cognitive impairment after coronary artery bypass grafting. J Neurol Sci.

[15] Gasparovic H, Borojevic M, Malojcic B, Gasparovic K, Biocina B (2013). Single aortic clamping in coronary artery bypass surgery reduces cerebral embolism and improves neurocognitive outcomes. Vasc Med.

[16] Gaudet JG, Meyers PM, McKinsey JF, Lavine SD, Gray W, Mitchell E, Connolly ES, Heyer EJ (2009). Incidence of moderate to severe cognitive dysfunction in patients treated with carotid artery stenting. Neurosurgery.

[17] Mok V, Ding D, Fu J, Xiong Y, Chu WW, Wang D, Abrigo JM, Yang J, Wong A, Zhao Q, Guo Q, Hong Z, Wong KS (2012). Transcranial Doppler ultrasound for screening cerebral small vessel disease: a community study. Stroke.

[18] Heliopoulos I, Artemis D, Vadikolias K, Tripsianis G, Piperidou C, Tsivgoulis G (2012). Association of ultrasonographic parameters with subclinical white-matter hyperintensities in hypertensive patients. Cardiovasc Psychiatry Neurol.

[19] Bakker SL, de Leeuw FE, de Groot JC, Hofman A, Koudstaal PJ, Breteler MM (1999). Cerebral vasomotor reactivity and cerebral white matter lesions in the elderly. Neurology.

[20] Fu JH, Lu CZ, Hong Z, Dong Q, Ding D, Wong KS (2006). Relationship between cerebral vasomotor reactivity and white matter lesions in elderly subjects without large artery occlusive disease. J Neuroimaging.

[21] Sorond FA, Kiely DK, Galica A, Moscufo N, Serrador JM, Iloputaife I, Egorova S, Dell’Oglio E, Meier DS, Newton E, Milberg WP, Guttmann CR, Lipsitz LA (2011). Neurovascular coupling is impaired in slow walkers: the MOBILIZE Boston Study. Ann Neurol.

[22] Sorond FA, Hollenberg NK, Panych LP, Fisher ND (2010). Brain blood flow and velocity: correlations between magnetic resonance imaging and transcranial Doppler sonography. J Ultrasound Med.

[23] Rosengarten B, Deppe M, Kaps M, Klingelhofer J (2012). Methodological aspects of functional transcranial Doppler sonography and recommendations for simultaneous EEG recording. Ultrasound Med Biol.

[24] Silvestrini M, Paolino I, Vernieri F, Pedone C, Baruffaldi R, Gobbi B, Cagnetti C, Provinciali L, Bartolini M (2009). Cerebral hemodynamics and cognitive performance in patients with asymptomatic carotid stenosis. Neurology.

[25] Silvestrini M, Vernieri F, Pasqualetti P, Matteis M, Passarelli F, Troisi E, Caltagirone C (2000). Impaired cerebral vasoreactivity and risk of stroke in patients with asymptomatic carotid artery stenosis. JAMA.

[26] Chen Y, Wolk DA, Reddin JS, Korczykowski M, Martinez PM, Musiek ES, Newberg AB, Julin P, Arnold SE, Greenberg JH, Detre JA (2011). Voxel-level comparison of arterial spin-labeled perfusion MRI and FDG-PET in Alzheimer disease. Neurology.

[27] Tosun D, Schuff N, Jagust W, Weiner MW (2016). Alzheimer’s Disease Neuroimaging Initiative. Discriminative power of arterial spin labeling magnetic resonance imaging and 18F-fluorodeoxyglucose positron emission tomography changes for amyloid-β-positive subjects in the Alzheimer’s disease continuum. Neurodegener Dis.

[28] Kyrtsos CR, Baras JS (2015). Modeling the role of the glymphatic pathway and cerebral blood vessel properties in Alzheimer’s disease pathogenesis. PLoS One.

[29] Moher D, Liberati A, Tetzlaff J, Altman DG (2009). Preferred reporting items for systematic reviews and meta-analyses: The PRISMA statement. Ann Intern Med.

[30] Aaslid R, Markwalder TM, Nornes H (1982). Noninvasive transcranial Doppler ultrasound recording of flow velocity in basal cerebral arteries. J Neurosurg.

[31] Topcuoglu MA (2012). Transcranial Doppler ultrasound in neurovascular diseases: diagnostic and therapeutic aspects. J Neurochem.

[32] Panerai RB, White RP, Markus HS, Evans DH (1998). Grading of cerebral dynamic autoregulation from spontaneous fluctuations in arterial blood pressure. Stroke.

[33] Tiecks FP, Lam AM, Matta BF, Strebel S, Douville C, Newell DW (1995). Effects of the Valsalva maneuver on cerebral circulation in healthy adults. A transcranial Doppler study. Stroke.

[34] Ainslie PN, Ashmead JC, Ide K, Morgan BJ, Poulin MJ (2005). Differential responses to CO2 and sympathetic stimulation in the cerebral and femoral circulations in humans. J Physiol.

[35] Poulin MJ, Robbins PA (1996). Indexes of flow and cross-sectional area of the middle cerebral artery using Doppler ultrasound during hypoxia and hypercapnia in humans. Stroke.

[36] Aaslid R (1987). Visually evoked dynamic blood flow response of the human cerebral circulation. Stroke.

[37] Stroobant N, Vingerhoets G (2001). Test-retest reliability of functional transcranial Doppler ultrasonography. Ultrasound Med Biol.

[38] Panerai RB, Eyre M, Potter JF (2012). Multivariate modeling of cognitive-motor stimulation on neurovascular coupling: transcranial Doppler used to characterize myogenic and metabolic influences. Am J Physiol Regul Integr Comp Physiol.

[39] Gosling RG, King DH (1974). Arterial assessment by Doppler-shift ultrasound. Proc R Soc Med.

[40] Keage HA, Churches OF, Kohler M, Pomeroy D, Luppino R, Bartolo ML, Elliott S (2012). Cerebrovascular function in aging and dementia: a systematic review of transcranial Doppler studies. Dement Geriatr Cogn Dis Extra.

[41] Bakker SL, de Leeuw FE, den Heijer T, Koudstaal PJ, Hofman A (2004). Cerebral haemodynamics in the elderly: the Rotterdam Study. Neuroepidemiology.

[42] Sattel H, Forstl H, Biedert S (1996). Senile dementia of Alzheimer type and multi-infarct dementia investigated by transcranial Doppler sonography. Dementia.

[43] Heun R, Knappertz V, Kraemer G (1994). Transcranial Doppler sonography in dementia of Alzheimer type. Dementia.

[44] van Beek AH, Lagro J, Olde-Rikkert MG, Zhang R, Claassen JA (2012). Oscillations in cerebral blood flow and cortical oxygenation in Alzheimer’s disease. Neurobiol Aging.

[45] Gur AY, Gücüyener D, Korczyn AD, Züner N, Gilutz Y, Özdemir G, Bornstein NM (2010). Cerebral vasomotor reactivity and dementia after ischemic stroke. Acta Neurol Scand.

[46] van Beek AH, Sijbesma JC, Jansen RW, Rikkert MG, Claassen JA (2010). Cortical oxygen supply during postural hypotension is further decreased in Alzheimer’s disease, but unrelated to cholinesterase inhibitor use. J Alzheimers Dis.

[47] Roher A, Garami Z, Alexandrov A, Kokjohn T, Esh C, Kalback W, Vedders L, Wilson J, Sabbagh M, Beach T (2006). Interaction of cardiovascular disease and neurodegeneration: transcranial Doppler ultrasonography and Alzheimer’s disease. Neurol Res.

[48] Biedert S, Förstl H, Hewer W (1995). Multiinfarct dementia versus Alzheimer’s disease: sonographic criteria. Angiology.

[49] Foerstl H, Biedert S, Hewer W (1989). Multiinfarct and Alzheimer-type dementia investigated by transcranial Doppler sonography. Biol Psychiatry.

[50] Asil T, Uzuner N (2005). Differentiation of vascular dementia and Alzheimer disease: a functional transcranial Doppler ultrasonographic study. J Ultrasound Med.

[51] Lee ST, Jung KH, Lee YS (2007). Decreased vasomotor reactivity in Alzheimer’s disease. J Clin Neurology.

[52] Biedert S, Förstl H, Hewer W (1993). The value of transcranial Doppler sonography in the differential diagnosis of Alzheimer disease versus multi-infarct dementia. Mol Chem Neuropathol.

[53] Roher AE, Garami Z, Tyas SL, Maarouf CL, Kokjohn TA, Belohlavek M, Vedders LJ, Connor D, Sabbagh MN, Beach TG, Emmerling MR (2011). Transcranial Doppler ultrasound blood flow velocity and pulsatility index as systemic indicators for Alzheimer’s disease. Alzheimers Dement.

[54] Kong XD, Zhang Y, Liu L, Sun N, Zhang MY, Zhag JN (2011). Endothelial progenitor cells with Alzheimer’s disease. Chin Med J.

[55] Stefani A, Sancesario G, Pierantozzi M, Leone G, Galati S, Hainsworth AH, Diomedi M (2009). CSF biomarkers, impairment of cerebral hemodynamics and degree of cognitive decline in Alzheimer’s and mixed dementia. J Neurol Sci.

[56] Vicenzini E, Ricciardi M, Altieri M, Puccinelli F, Bonaffini N, Di Piero V, Lenzi G (2007). Cerebrovascular reactivity in degenerative and vascular dementia: a transcranial Doppler study. Eur Neurol.

[57] Franceschi M, Alberoni M, Bressi S, Canal N, Comi G, Fazio F, Grassi F, Perani D, Volonté MA (1995). Correlations between cognitive impairment, middle cerebral artery flow velocity and cortical glucose metabolism in the early phase of Alzheimer’s disease. Dementia.

[58] Caamaño J, Gómez M, Cacabelos R (1993). Transcranial Doppler ultrasonography in senile dementia: neuropsychological correlations. Methods Find Exp Clin Pharmacol.

[59] Gucuyener DO, Yenilmez C, Ayranci U, Ozdemir F, Uzuner N, Ozkan S, Kaptanoglu C, Ozdemir G (2010). An analysis of changes in cerebral blood flood velocities in depressive pseudo-dementia and Alzheimer disease patients. Neurologist.

[60] Provinciali L, Minicotti P, Ceravolo G, Angeleri F, Sanguinetti CN (1990). Transcranial Doppler sonography as a diagnostic tool in vascular dementia. Eur Neurol.

[61] Sabayan B, Jansen S, Oleksik AM, van Osch MJ, van Buchem MA, van Vliet P, de Craen AJ, Westendorp RG (2012). Cerebrovascular hemodynamics in Alzheimer's disease and vascular dementia: a meta-analysis of transcranial Doppler studies. Ageing Res Rev.

[62] Likitjaroen Y, Suwanwela NC, Phanthumchinda K (2009). Vasoreactivity induced by acetazolamide in patients with vascular dementia versus Alzheimer’s disease. J Neurol Sci.

[63] Ries F, Horn R, Hillekamp J, Honisch C, Konig M, Solymosi L (1993). Differentiation of multi-infarct and Alzheimer dementia by intracranial hemodynamic parameters. Stroke.

[64] Anzola GP, Galluzzi S, Mazzucco S, Frisoni GB (2011). Autonomic dysfunction in mild cognitive impairment: a transcranial Doppler study. Acta Neurol Scand.

[65] Lupetin AR, Davis DA, Beckman I, Dash N (1995). Transcranial Doppler sonography. Part 1. Principles, technique, and normal appearances. Radiographics.

[66] Martin PJ, Evans DH, Naylor AR (1995). Measurement of blood flow velocity in the basal cerebral circulation: advantages of transcranial color-coded sonography over conventional transcranial Doppler. J Clin Ultrasound.

[67] Matteis M, Bivona U, Catani S, Pasqualetti P, Formisano R, Vernieri F (2009). Functional transcranial Doppler assessment of cerebral blood flow velocities changes during attention tasks. Eur J Neurol.

[68] Knecht S, Deppe M, Ebner A, Henningsen H, Huber T, Jokeit H (1998). Noninvasive determination of language lateralization by functional transcranial Doppler sonography: A comparison with the Wada test. Stroke.

[69] Knake S, Haag A, Hamer HM, Dittmer C, Bien S, Oertel WH (2003). Language lateralization in patients with temporal lobe epilepsy: A comparison of functional transcranial Doppler sonography and the Wada test. Neuroimage.

[70] Azevedo E, Mendes A, Seixas D, Santos R, Castro P, Ayres-Basto M (2012). Functional transcranial Doppler: Presymptomatic changes in Fabry disease. Eur Neurol.

[71] Rosengarten B, Dost A, Kaufmann A, Gortner L, Kaps M (2002). Impaired cerebrovascular reactivity in type 1 diabetic children. Diabetes Care.

[72] Rosengarten B, Gruessner S, Aldinger C, Künzel W, Kaps M (2004). Abnormal regulation of maternal cerebral blood flow under conditions of gestational diabetes mellitus. Ultraschall Med.

[73] Kolodny E, Fellgiebel A, Hilz MJ, Sims K, Caruso P, Phan TG (2015). Cerebrovascular involvement in Fabry disease: Current status of knowledge. Stroke.

[74] Rosengarten B, Aldinger C, Spiller A, Kaps M (2003). Neurovascular coupling remains unaffected during normal aging. J Neuroimaging.

[75] Sorond FA, Schnyer DM, Serrador JM, Milberg WP, Lipsitz LA (2008). Cerebral blood flow regulation during cognitive tasks: effects of healthy aging. Cortex.

[76] Sorond FA, Hurwitz S, Salat DH, Greve DN, Fisher ND (2013). Neurovascular coupling, cerebral white matter integrity, and response to cocoa in older people. Neurology.

[77] Rosengarten B, Paulsen S, Burr O, Kaps M (2009). Neurovascular coupling in Alzheimer patients: effect of acetylcholine-esterase inhibitors. Neurobiol Aging.

[78] Azevedo E, Castro P, Santos R, Freitas J, Coelho T, Rosengarten B (2011). Autonomic dysfunction affects cerebral neurovascular coupling. Clin Auton Res.

[79] Azevedo E, Rosengarten B, Santos R, Freitas J, Kaps M (2007). Interplay of cerebral autoregulation and neurovascular coupling evaluated by functional TCD in different orthostatic conditions. J Neurol.

[80] Azevedo E, Santos R, Freitas J, Rosas MJ, Gago M, Garrett C (2010). Deep brain stimulation does not change neurovascular coupling in non-motor visual cortex: An autonomic and visual evoked blood flow velocity response study. Parkinsonism Relat Disord.

[81] Silvestrini M, Troisi E, Matteis M, Cupini LM, Caltagirone C (1995). Involvement of the healthy hemisphere in recovery from aphasia and motor deficit in patients with cortical ischemic infarction: A transcranial Doppler study. Neurology.

[82] Salinet AS, Robinson TG, Panerai RB (2013). Active, passive, and motor imagery paradigms: Component analysis to assess neurovascular coupling. J Appl Physiol (1985).

[83] Boban M, Crnac P, Junakovic A, Garami Z, Malojcic B (2014). Blood flow velocity changes in anterior cerebral arteries during cognitive tasks performance. Brain Cogn.

[84] Badcock NA, Holt G, Holden A, Bishop DV (2012). Doposcci: A functional transcranial Doppler ultrasonography summary suite for the assessment of cerebral lateralization of cognitive function. J Neurosci Methods.

[85] Deppe M, Knecht S, Lohmann H, Ringelstein EB (2004). A method for the automated assessment of temporal characteristics of functional hemispheric lateralization by transcranial Doppler sonography. J Neuroimaging.

[86] Cupini LM, Matteis M, Troisi E, Sabbadini M, Bernardi G, Caltagirone C (1996). Bilateral simultaneous transcranial Doppler monitoring of flow velocity changes during visuospatial and verbal working memory tasks. Brain.

[87] Boban M, Crnac P, Junakovic A, Malojcic B (2014). Hemodynamic monitoring of middle cerebral arteries during cognitive tasks performance. Psychiatry Clin Neurosci.

[88] Rosengarten B, Huwendiek O, Kaps M (2001). Neurovascular coupling in terms of a control system: validation of a second-order linear system model. Ultrasound Med Biol.

[89] Garnham J, Panerai RB, Naylor AR, Evans DH (1999). Cerebrovascular response to dynamic changes in pCO2. Cerebrovasc Dis.

[90] Ulrich PT, Becker T, Kempski OS (1995). Correlation of cerebral blood flow and MCA flow velocity measured in healthy volunteers during acetazolamide and CO2 stimulation. J Neurol Sci.

[91] Dahl A, Russell D, Rootwelt K, Nyberg-Hansen R, Kerty E (1995). Cerebral vasoreactivity assessed with transcranial Doppler and regional cerebral blood flow measurements. Dose, serum concentration, and time course of the response to acetazolamide. Stroke.

[92] Piepgras A, Schmiedek P, Leinsinger G, Haberl RL, Kirsch CM, Einhäupl KM (1990). A simple test to assess cerebrovascular reserve capacity using transcranial Doppler sonography and acetazolamide. Stroke.

[93] Hauge A, Nicolaysen G, Thoresen M (1983). Acute effects of acetazolamide on cerebral blood flow in man. Acta Physiol Scand.

[94] Maren TH (1967). Carbonic anhydrase: chemistry, physiology, and inhibition. Physiol Rev.

[95] Vorstrup S, Henriksen L, Paulson OB (1984). Effect of acetazolamide on cerebral blood flow and cerebral metabolic rate for oxygen. J Clin Invest.

[96] Lavi S, Gaitini D, Milloul V, Jacob G (2006). Impaired cerebral CO2 vasoreactivity: association with endothelial dysfunction. Am J Physiol Heart Circ Physiol.

[97] Matteis M, Silvestrini M, Troisi E, Bragoni M, Vernieri F, Caltagirone C (1998). Cerebral hemodynamic patterns during stimuli tasks in multi-infarct and Alzheimer types of dementia. Acta Neurol Scand.

[98] Silvestrini M, Pasqualetti P, Baruffaldi R, Bartolini M, Handouk Y, Matteis M, Moffa F, Provinciali L, Vernieri F (2006). Cerebrovascular reactivity and cognitive decline in patients with Alzheimer disease. Stroke.

[99] Buratti L, Balestrini S, Altamura C, Viticchi G, Falsetti L, Luzzi S, Provinciali L, Vernieri F, Silvestrini M (2015). Markers for the risk of progression from mild cognitive impairment to Alzheimer’s disease. J Alzheimers Dis.

[100] Shim Y, Yoon B, Shim DS, Kim W, An JY, Yang DW (2015). Cognitive correlates of cerebral vasoreactivity on transcranial Doppler in older adults. J Stroke Cerebrovasc Dis.

[101] Viticchi G, Falsetti L, Vernieri F, Altamura C, Bartolini M, Luzzi S, Provinciali L, Silvestrini M (2012). Vascular predictors of cognitive decline in patients with mild cognitive impairment. Neurobiol Aging.

[102] Viticchi G, Falsetti L, Vernieri F, Altamura C, Altavilla R, Luzzi S, Bartolini M, Provinciali L, Silvestrini M (2014). Apolipoprotein E genotype and cerebrovascular alterations can influence conversion to dementia in patients with mild cognitive impairment. J Alzheimers Dis.

[103] Zavoreo I, Kes VB, Morovic S, Seric V, Demarin V (2010). Breath holding index in detection of early cognitive decline. J Neurol Sci.

[104] Zavoreo I, Demarin V (2010). Breath holding index and arterial stiffness as markers of vascular aging. Curr Aging Sci.

[105] Balestrini S, Perozzi C, Altamura C, Vernieri F, Luzzi S, Bartolini M, Provinciali L, Silvestrini M (2013). Severe carotid stenosis and impaired cerebral hemodynamics can influence cognitive deterioration. Neurology.

[106] Markus HS, Harrison MJG (1992). Estimation of cerebrovascular reactivity using transcranial Doppler, including the use of breath-holding as the vasodilatory stimulus. Stroke.

[107] Ringelstein EB, Sievers C, Ecker S, Schneider PA, Otis SM (1988). Non invasive assessment of CO2 induced cerebral vasomotor response in normal individuals and patients with internal carotid artery occlusions. Stroke.

[108] Bishop CCR, Powell S, Rutt D, Browse NL (1986). Transcranial Doppler measurement of middle cerebral artery blood flow velocity: a validation study. Stroke.

[109] Maeda H, Matsumoto M, Handa N (1994). Reactivity of cerebral blood flow to carbon dioxide in hypertensive patients: evaluation by the transcranial Doppler method. J Hypertens.

[110] Aaslid R, Lindegaard KF, Sorteberg W, Nornes H (1989). Cerebral autoregulation dynamics in humans. Stroke.

[111] Dawson SL, Blake MJ, Panerai RB, Potter JF (2000). Dynamic but not static cerebral autoregulation is impaired in acute ischaemic stroke. Cerebrovasc Dis.

[112] Tiecks FP, Douville C, Byrd S, Lam AM, Newell DW (1996). Evaluation of impaired cerebral autoregulation by the Valsalva maneuver. Stroke.

[113] Greenfield JC, Rembert JC, Tindall GT (1984). Transient changes in cerebral vascular resistance during the Valsalva maneuver in man. Stroke.

[114] Giller CA (1991). A bedside test for cerebral autoregulation using transcranial Doppler ultrasound. Acta Neurochir (Wien).

[115] Rätsep T, Asser T (2001). Cerebral hemodynamic impairment after aneurismal subarachnoid hemorrhage as evaluated using transcranial Doppler ultrasonography: relationship to delayed cerebral ischemia and clinical outcome. J Neurosurg.

[116] Sorond FA, Serrador JM, Jones RN, Shaffer ML, Lipsitz LA (2009). The sit-to-stand technique for the measurement of dynamic cerebral autoregulation. Ultrasound Med Biol.

[117] Tiecks FP, Lam AM, Aaslid R, Newell DW (1995). Comparison of static and dynamic cerebral autoregulation measurements. Stroke.

[118] Panerai RB, Evans DH, Naylor AR (1999). Influence of arterial blood pressure on cerebrovascular reactivity. Stroke.

[119] Diehl RR, Linden D, Lücke D, Berlit P (1995). Phase relationship between cerebral blood flow velocity and blood pressure. A clinical test of autoregulation. Stroke.

[120] Birch AA, Dirnhuber MJ, Hartley-Davies R, Iannotti F, Neil-Dwyer G (1995). Assessment of autoregulation by means of periodic changes in blood pressure. Stroke.

[121] Blaber AP, Bondar RL, Stein F, Dunphy PT, Moradshahi P, Kassam MS, Freeman R (1997). Complexity of middle cerebral artery blood flow velocity: effects of tilt and autonomic failure. Am J Physiol.

[122] Serrador JM, Sorond FA, Vyas M, Gagnon M, Iloputaife ID, Lipsitz LA (2005). Cerebral pressure-flow relations in hypertensive elderly humans: transfer gain in different frequency domains. J Appl Physiol.

[123] Lundar T, Lindegaard KF, Frøysaker T, Aaslid R, Grip A, Nornes H (1985). Dissociation between cerebral autoregulation and carbon dioxide reactivity during nonpulsatile cardiopulmonary bypass. Ann Thorac Surg.

[124] Paulson OB, Strandgaard S, Edvinsson L (1990). Cerebral autoregulation. Cerebrovasc Brain Metab Rev.

[125] Niwa K, Kazama K, Younkin L, Younkin SG, Carlson GA, Iadecola C (2002). Cerebrovascular autoregulation is profoundly impaired in mice overexpressing amyloid precursor protein. Am J Physiol Heart Circ Physiol.

[126] Purkayastha S, Fadar O, Mehregan A, Salat DH, Moscufo N, Meier DS, Guttmann CR, Fisher ND, Lipsitz LA, Sorond FA (2014). Impaired cerebrovascular hemodynamics are associated with cerebral white matter damage. J Cereb Blood Flow Metab.

[127] Brickman AM, Guzman VA, Gonzalez-Castellon M, Razlighi Q, Gu Y, Narkhede A, Janicki S, Ichise M, Stern Y, Manly JJ, Schupf N, Marshall RS (2015). Cerebral autoregulation, beta amyloid, and white matter hyperintensities are interrelated. Neurosci Lett.

[128] Claassen JA, Zhang R (2011). Cerebral autoregulation in Alzheimer’s disease. J Cereb Blood Flow Metab.

[129] Meel-van den Abeelen AS, van Beek AH, Slump CH, Panerai RB, Claassen JA (2014). Transfer function analysis for the assessment of cerebral autoregulation using spontaneous oscillations in blood pressure and cerebral blood flow. Med Eng Phys.

[130] Claassen JA, Meel-van den Abeelen AS, Simpson DM, Panerai RB, International Cerebral Autoregulation Research Network (CARNet) (2016). Transfer function analysis of dynamic cerebral autoregulation: A white paper from the International Cerebral Autoregulation Research Network. J Cereb Blood Flow Metab.

[131] Mitchell GF, van Buchem MA, Sigurdsson S (2011). Arterial stiffness, pressure and flow pulsatility and brain structure and function: the Age, Gene/Environment Susceptibility-Reykjavik study. Brain.

[132] Webb AJ, Simoni M, Mazzucco S, Kuker W, Schulz U, Rothwell PM (2012). Increased cerebral arterial pulsatility in patients with leukoaraiosis: arterial stiffness enhances transmission of aortic pulsatility. Stroke.

[133] Tanaka T, Shimizu T, Fukuhara T (2009). The relationship between leukoaraiosis volume and parameters of carotid artery duplex ultrasonographic scanning in asymptomatic diabetic patients. Comput Med Imaging Graph.

[134] Aribisala BS, Morris Z, Eadie E (2014). Blood pressure, internal carotid artery flow parameters, and age-related white matter hyperintensities. Hypertension.

[135] Turk M, Zupan M, Zaletel M, Žvan B, Pretnar OJ (2015). Carotid arterial hemodynamic in ischemic leukoaraiosis suggests hypoperfusion mechanism. Eur Neurol.

[136] Schreiber SJ, Doepp F, Spruth E, Kopp UA, Valdueza JM (2005). Ultrasonographic measurement of cerebral blood flow, cerebral circulation time and cerebral blood volume in vascular and Alzheimer’s dementia. J Neurol.

[137] Berman SE, Wang X, Mitchell CC, Kundu B, Jackson DC, Wilbrand SM, Varghese T, Hermann BP, Rowley HA, Johnson SC, Dempsey RJ (2015). The relationship between carotid artery plaque stability and white matter ischemic injury. Neuroimage Clin.

[138] Casado-Naranjo I, Romero Sevilla R, Portilla Cuenca JC, Duque de San Juan B, Calle Escobar ML, Fernández Pereira L, Fuentes JM, Ramírez-Moreno JM (2016). Association between subclinical carotid atherosclerosis, hyperhomocysteinaemia and mild cognitive impairment. Acta Neurol Scand.

[139] Knopman DS, Penman AD, Catellier DJ, Coker LH, Shibata DK, Sharrett AR, Mosley TH (2011). Vascular risk factors and longitudinal changes on brain MRI: the ARIC study. Neurology.

[140] Shrestha I, Takahashi T, Nomura E, Ohtsuki T, Ohshita T, Ueno H, Kohriyama T, Matsumoto M (2009). Association between central systolic blood pressure, white matter lesions in cerebral MRI and carotid atherosclerosis. Hypertens Res.

[141] Touboul PJ, Hennerici MG, Meairs S, Adams H, Amarenco P, Bornstein N, Csiba L, Desvarieux M, Ebrahim S, Hernandez Hernandez R, Jaff M, Kownator S, Naqvi T, Prati P, Rundek T, Sitzer M, Schminke U, Tardif JC, Taylor A, Vicaut E, Woo KS (2012). Mannheim carotid intima-media thickness and plaque consensus (2004-2006-2011). An update on behalf of the advisory board of the 3rd, 4th and 5th Watching the Risk Symposia, at the 13th, 15th and 20th European Stroke Conferences, Mannheim, Germany, 2004, Brussels, Belgium, 2006, and Hamburg, Germany, 2011. Cerebrovasc Dis.

[142] Poels MM, Zaccai K, Verwoert GC (2012). Arterial stiffness and cerebral small vessel disease: the Rotterdam Scan Study. Stroke.

[143] Turk M, Zaletel M, Pretnar-Oblak J (2016). Ratio between carotid artery stiffness and blood flow - a new ultrasound index of ischemic leukoaraiosis. Clin Interv Aging.

[144] Henskens LH, Kroon AA, van Oostenbrugge RJ (2008). Increased aortic pulse wave velocity is associated with silent cerebral small-vessel disease in hypertensive patients. Hypertension.

[145] Hatanaka R, Obara T, Watabe D (2011). Association of arterial stiffness with silent cerebrovascular lesions: The Ohasama study. Cerebrovasc Dis.

[146] Nation DA, Preis SR, Beiser A, Bangen KJ, Delano-Wood L, Lamar M, Libon DJ, Seshadri S, Wolf PA, Au R (2016). Pulse pressure is associated with early brain atrophy and cognitive decline: modifying effects of APOE-ε4. Alzheimer Dis Assoc Disord.

[147] Laurent S, Cockcroft J, Van Bortel L (2006). Expert consensus document on arterial stiffness: methodological issues and clinical applications. Eur Heart J.

[148] Paini A, Boutouyrie P, Calvet D, Tropeano AI, Laloux B, Laurent S (2006). Carotid and aortic stiffness: determinants of discrepancies. Hypertension.

[149] Turk M, Pretnar-Oblak J, Zupan M, Zvan B, Zaletel M (2015). Ultrasound diagnostics of carotid arterial stiffness in patients with ischemic leukoaraiosis. Ultrasound Med Biol.

[150] Brisset M, Boutouyrie P, Pico F (2013). Large-vessel correlates of cerebral small-vessel disease. Neurology.

[151] Meinders JM, Hoeks AP (2004). Simultaneous assessment of diameter and pressure waveforms in the carotid artery. Ultrasound Med Biol.

[152] Rhee MY, Lee HY, Park JB (2008). Measurements of arterial stiffness: methodological aspects. Korean Circ J.

[153] Jaroch J, Łoboz Grudzień K, Bociąga Z, Kowalska A, Kruszyńska E, Wilczyńska M, Dudek K (2012). The relationship of carotid arterial stiffness to left ventricular diastolic dysfunction in untreated hypertension. Kardiol Pol.

[154] Vermeer SE, Prins ND, den Heijer T, Hofman A, Koudstaal PJ, Breteler MM (2003). Silent brain infarcts and the risk of dementia and cognitive decline. N Engl J Med.

[155] Purandare N, Welsh S, Hutchinson S, Riding G, Burns A, McCollum C (2005). Cerebral emboli and paradoxical embolisation in dementia: a pilot study. Int J Geriatr Psychiatry.

[156] Purandare N, Burns A, Daly KJ, Hardicre J, Morris J, Macfarlane G, McCollum C (2006). Cerebral emboli as a potential cause of Alzheimer’s disease and vascular dementia: case-control study. BMJ.

[157] Purandare N, Voshaar RC, Morris J, Byrne JE, Wren J, Heller RF, McCollum CN, Burns A (2007). Asymptomatic spontaneous cerebral emboli predict cognitive and functional decline in dementia. Biol Psychiatry.

[158] Purandare N, Burns A, Morris J, Perry E, Wren J, McCollum C (2012). Association of cerebral emboli with accelerated cognitive deterioration in Alzheimer’s disease and vascular dementia. Am J Psychiatry.

[159] Droste DW, Markus HS, Brown MM (1994). The effect of different settings of ultrasound pulse amplitude, gain and sample volume on the appearance of emboli studied in a transcranial Doppler model. Cerebrovasc Dis.

[160] Ringelstein EB, Droste DW, Babikian VL, Evans DH, Grosset DG, Kaps M, Markus HS, Russell D, Siebler M (1998). Consensus on microembolus detection by TCD. International Consensus Group on Microembolus Detection. Stroke.

[161] Garami ZF, Bismuth J, Charlton-Ouw KM, Davies MG, Peden EK, Lumsden AB (2009). Feasibility of simultaneous pre- and postfilter transcranial Doppler monitoring during carotid artery stenting. J Vasc Surg.

[162] Yan L, Liu CY, Smith RX, Jog M, Langham M, Krasileva K (2016). Assessing intracranial vascular compliance using dynamic arterial spin labeling. Neuroimage.

[163] Zaharchuk G, El Mogy IS, Fischbein NJ, Albers GW (2012). Comparison of arterial spin labeling and bolus perfusion-weighted imaging for detecting mismatch in acute stroke. Stroke.

[164] Bokkers RP, Hernandez DA, Merino JG, Mirasol RV, van Osch MJ, Hendrikse J, Warach S, Latour LL, National Institutes of Health Stroke Natural History Investigators (2012). Whole-brain arterial spin labeling perfusion MRI in patients with acute stroke. Stroke.

[165] Altrichter S, Kulcsar Z, Jägersberg M, Federspiel A, Viallon M, Schaller K, Rüfenacht DA, Lövblad KO (2009). Arterial spin labeling shows cortical collateral flow in the endovascular treatment of vasospasm after post-traumatic subarachnoid hemorrhage. J Neuroradiol.

[166] Gao YZ, Zhang JJ, Liu H, Wu GY, Xiong L, Shu M (2013). Regional cerebral blood flow and cerebrovascular reactivity in Alzheimer’s disease and vascular dementia assessed by arterial spinlabeling magnetic resonance imaging. Curr Neurovasc Res.

[167] Firbank MJ, He J, Blamire AM, Singh B, Danson P, Kalaria RN, O’Brien JT (2011). Cerebral blood flow by arterial spin labeling in poststroke dementia. Neurology.

[168] Du AT, Jahng GH, Hayasaka S, Kramer JH, Rosen HJ, Gorno-Tempini ML, Rankin KP, Miller BL, Weiner MW, Schuff N (2006). Hypoperfusion in frontotemporal dementia and Alzheimer disease by arterial spin labeling MRI. Neurology.

[169] Binnewijzend MA, Kuijer JP, Benedictus MR, van der Flier WM, Wink AM, Wattjes MP, van Berckel BN, Scheltens P, Barkhof F (2013). Cerebral blood flow measured with 3D pseudocontinuous arterial spin-labeling MR imaging in Alzheimer disease and mild cognitive impairment: a marker for disease severity. Radiology.

[170] Montagne A, Nation DA, Pa J, Sweeney MD, Toga AW, Zlokovic BV (2016). Brain imaging of neurovascular dysfunction in Alzheimer’s disease. Acta Neuropathol.

[171] Musiek ES, Chen Y, Korczykowski M, Saboury B, Martinez PM, Reddin JS, Alavi A, Kimberg DY, Wolk DA, Julin P, Newberg AB, Arnold SE, Detre JA (2012). Direct comparison of fluorodeoxyglucose positron emission tomography and arterial spin labeling magnetic resonance imaging in Alzheimer’s disease. Alzheimers Dement.

[172] Grade M, Hernandez Tamames JA, Pizzini FB, Achten E, Golay X, Smits M (2015). A neuroradiologist's guide to arterial spin labeling MRI in clinical practice. Neuroradiology.

[173] Østergaard L, Aamand R, Gutiérrez-Jiménez E, Ho YC, Blicher JU, Madsen SM, Nagenthiraja K, Dalby RB, Drasbek KR, Møller A, Brændgaard H, Mouridsen K, Jespersen SN, Jensen MS, West MJ (2013). The capillary dysfunction hypothesis of Alzheimer’s disease. Neurobiol Aging.

[174] Xekardaki A, Rodriguez C, Montandon ML, Toma S, Tombeur E, Herrmann FR, Zekry D, Lovblad KO, Barkhof F, Giannakopoulos P, Haller S (2015). Arterial spin labeling may contribute to the prediction of cognitive deterioration in healthy elderly individuals. Radiology.

[175] Assländer J, Zahneisen B, Hugger T, Reisert M, Lee HL, LeVan P, Hennig J (2013). Single shot whole brain imaging using spherical stack of spirals trajectories. Neuroimage.

[176] Alsop DC, Detre JA, Golay X, Günther M, Hendrikse J, Hernandez-Garcia L (2015). Recommended implementation of arterial spin-labeled perfusion MRI for clinical applications: a consensus of the ISMRM perfusion study group and the European consortium for ASL in dementia. Magn Reson Med.

[177] Amukotuwa SA, Yu C, Zaharchuk G (2016). 3D pseudocontinuous arterial spin labeling in routine clinical practice: a review of clinically significant artifacts. J Magn Reson Imaging.

[178] Wu WC, Lin SC, Wang DJ, Chen KL, Li YD (2013). Measurement of cerebral white matter perfusion using pseudocontinuous arterial spin labeling 3T magnetic resonance imaging--an experimental and theoretical investigation of feasibility. PLoS One.

[179] Fisse AL, Pueschel J, Deppe M, Ringelstein EB, Ritter MA (2016). TCD-profiling using AVERAGE. A new technique to evaluate transcranial Doppler ultrasound flow spectra of subjects with cerebral small vessel disease. Cerebrovasc Dis.

[180] Morovic S, Jurasic MJ, Martinic Popovic I, Seric V, Lisak M, Demarin V (2009). Vascular characteristics of patients with dementia. J Neurol Sci.

[181] Jurasic MJ, Popovic IM, Morovic S, Trkanjec Z, Seric V, Demarin V (2009). Can beta stiffness index be proposed as risk factor for dementia. J Neurol Sci.

[182] Kearney-Schwartz A, Rossignol P, Bracard S (2009). Vascular structure and function is correlated to cognitive performance and white matter hyperintensities in older hypertensive patients with subjective memory complaints. Stroke.

